# Q-factor enhancement of Fano resonance in all-dielectric metasurfaces by modulating meta-atom interactions

**DOI:** 10.1038/s41598-017-07715-6

**Published:** 2017-08-15

**Authors:** Guanghou Sun, Lierong Yuan, Yi Zhang, Xuejin Zhang, Yongyuan Zhu

**Affiliations:** 1 National Laboratory of Solid State Microstructures and Collaborative Innovation Center of Advanced Microstructures and Key Laboratory of Modern Acoustics and School of Physics, Nanjing University, Nanjing, 210093 China; 2 College of Engineering and Applied Sciences, Nanjing University, Nanjing, 210093 China; 3 School of Science, Jiujiang University, Jiujiang, 332005 China

## Abstract

We numerically investigated the effects of meta-atom interactions on the Fano resonance in all-dielectric metasurfaces by introducing alternately flipped asymmetric paired bars (APBs) and split asymmetric paired bars (SAPBs). With alternately flipped configurations, the Q-factor of the Fano resonance is significantly enhanced up to one order of magnitude, and the electric field is strengthened by more than twice. Abnormally, the Q-factor increases with gap size in the alternately flipped SAPBs. These are attributed to the destructive interaction among nearest-neighbor dipole resonators. The Q-factor of 10^8^ and Raman enhancement factor of 10^9^ in the gap can be realized with the alternately flipped SAPBs made of Si. Our study provides a way to improve performance of practical devices such as ultrasensitive sensors, nonlinear optics, and quantum emitters.

## Introduction

Engineering high quality factor (Q-factor) resonant responses is an active topic in the field of metamaterials^1, 2^. High Q-factor resonators are required to achieve a significant field confinement and low loss, thus providing an efficient platform for strong light-matter interactions in various fields of applications, such as nonlinear optics^3^, surface-enhanced Raman scattering^4^, plasmonic lasers^5^, photoswitching^6^, chiral optical response^7^, induced doping of graphene^8^ and sensors^9, 10^. The Fano resonance (FR) is one of the effective methods to realize the high Q-factor resonance in metamaterials because the radiation loss is suppressed. Such resonance generally originates from the interference of a broad bright mode and a narrow dark mode, showing an asymmetric spectral lineshape with a narrow dip. FRs can be supported by coupled metallic nanostructures^1, 2, 7, 11–15^. The performance of FR-based devices is primarily determined by their Q-factor and spectral contrast. Because a dark mode has nearly zero net dipole moment and small radiation loss, the Q-factor of the FR is generally larger than that of the dipole resonance.

The disadvantage of metallic metamaterials is the large inherent Ohmic loss in the visible and near-infrared wavelength ranges, which limits the Q-factor about the order of ten. This disadvantage has severely impeded many applications of the FR. One promising route to achieve high Q-factor FR is based on low-loss all-dielectric resonators whose properties result from Mie resonances^16, 17^. Recently, theoretical and experimental studies show that the sharp FR can also be achieved by exciting dark modes in all-dielectric metamaterials^18–20^. The meta-atoms can be arranged into periodic array to form metamaterials, where the electromagnetic properties result from individual properties and the collective responses. The interactions among meta-atoms have great influence upon the Q-factor. Effects of different relative configurations of unit cells on the Q-factor of resonances have been studied in metallic metamaterials^15, 21–23^, which have not been taken into account in all-dielectric metamaterials yet.

In this study, we numerically investigated the effects of meta-atom interactions on electromagnetic properties in all-dielectric metasurfaces made of Si with different configurations of unit cells. The Q-factor, as well as the electric field of FRs in alternately flipped asymmetric paired bars (APBs) and split asymmetric paired bars (SAPBs) is significantly larger than that in non-flipped APBs and SAPBs. Most interestingly, the broader the gap, the larger the Q-factor in alternately flipped SAPBs. And the less the absorption loss is, the more the Q-factor of the FR can be enhanced.

## Results and Discussion

**Figure 1:**
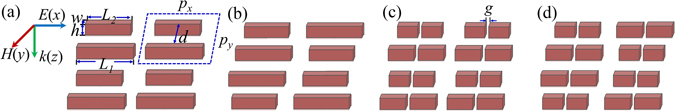
Schematics of dielectric metasurfaces comprising periodical arrays of (**a**) APBs, (**b**) alternately flipped APBs, (**c**) SAPBs and (**d**) alternately flipped SAPBs with gaps in the middle.

The schematic of all-dielectric metasurfaces is shown in Fig. 1. Figure 1(a) shows an all-dielectric metasurface comprising periodical array of APBs. The unit cell of the APBs is composed of two Si bars with slightly different lengths. The paired bars are positioned in parallel with the spacing *d* = 200 nm. The length of the long bar *L*_1_ is fixed to be 750 nm, and the length of the short bar *L*_2_ is altered. The width (*w*) and height (*h*) of the two bars are 200 nm and 150 nm, respectively. The lattice constants along both axes *p*_*x*_ and *p*_*y*_ are 900 nm. Figure 1(b) shows an all-dielectric metasurface composed of alternately flipped APBs, in which two APBs positioned on the second diagonal of 2 × 2 unit cells are flipped. Figure 1(c) and (d) are metasurfaces made of SAPBs and alternately flipped SAPBs with gaps *g* = 50 nm in the middle. In order to simulate the optical properties of the proposed metasurfaces, the frequency-domain finite element method^24^ is employed. Si is initially supposed to be lossless with a refractive index of *n* = 3.5, and the material loss is considered further. The surrounding medium is supposed to be air (*n* = 1). The incident plane wave has an electric field polarized along the *x* direction with a propagation vector along the *z* direction, as shown in Fig. 1.

**Figure 2:**
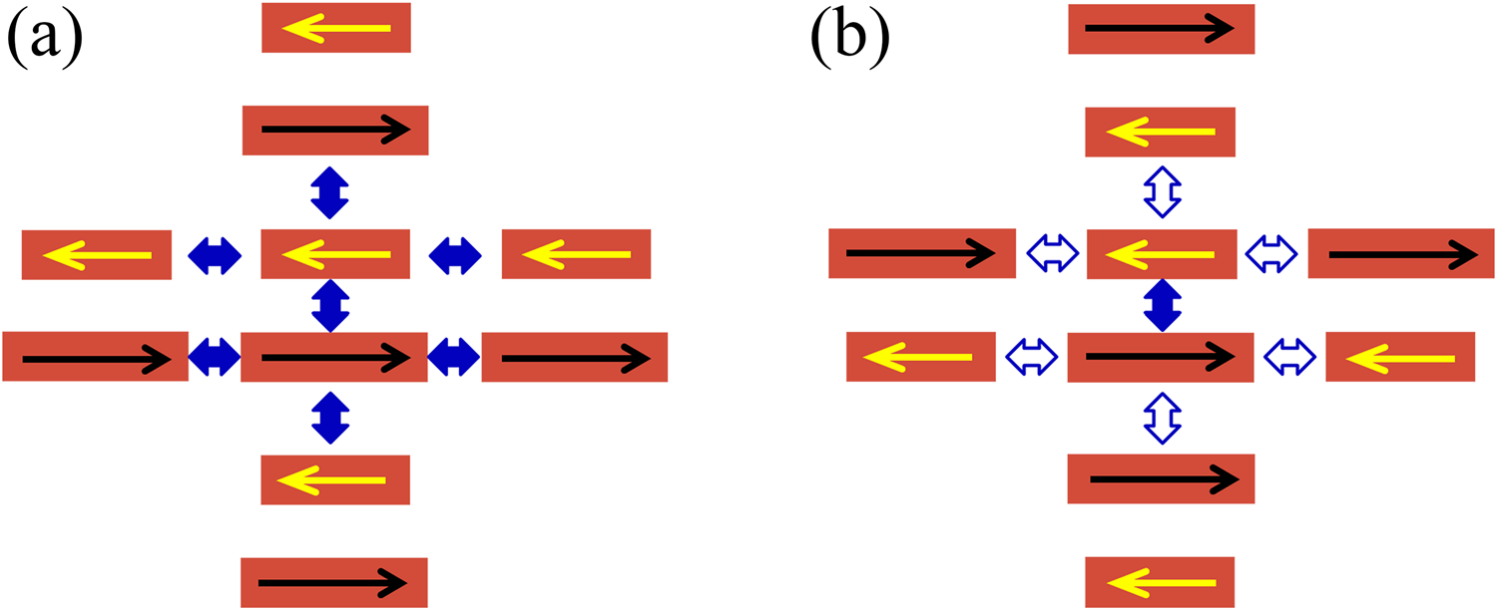
Nearest-neighbor dipole interactions in (**a**) non-flipped APBs and (**b**) alternately flipped APBs. Single-way arrows indicate electric dipole moments. Solid and hollow two-way arrows indicate constructive and destructive interactions, respectively.

When the electric field component of incident light parallels to the long axis of the Si bar, the corresponding component of the displacement current inside the bar increases dramatically. This resonance is similar to the electric dipole resonance in metallic metamaterials. Here the bar is also called the meta-atom. The dark mode is excited in a unit cell of the APBs, which is characterized by anti-phased displacement currents in the two Si bars of the unit cell^19^ (see Fig. 2). Each electric dipole is excited by not only the external electric field but also electric fields produced by neighboring meta-atoms. The meta-atom interactions are different when configurations of unit cells are changed. For non-flipped configuration, the dipole moment of a bar is constructively driven by nearest-neighbor dipoles, shown in Fig. 2(a). However, for alternately flipped configuration, the dipole moment of a bar is destructively driven by nearest-neighbor dipoles outside of the APBs, shown in Fig. 2(b).

Radiation loss is proportional to a net dipole moment per unit area *μ* in metamaterials, which at dark mode can be written as^23^

1 $$\mu =m\gamma ({\mu }_{l}-{\mu }_{s})$$

where *μ*_*l*_ and *μ*_*s*_ are magnitude of dipole moments in the independent long and short bars, respectively, *m* is the density of APBs/SAPBs per unit area, and *γ* is a constant related to the nearest-neighbor dipole interactions, which becomes smaller (larger) than 1 when dipole interactions are destructively (constructively). The *μ* in alternately flipped configuration becomes smaller than that in the non-flipped configuration. Therefore, radiation loss is suppressed in the alternately flipped configuration, which leads to a high Q-factor.

**Figure 3:**
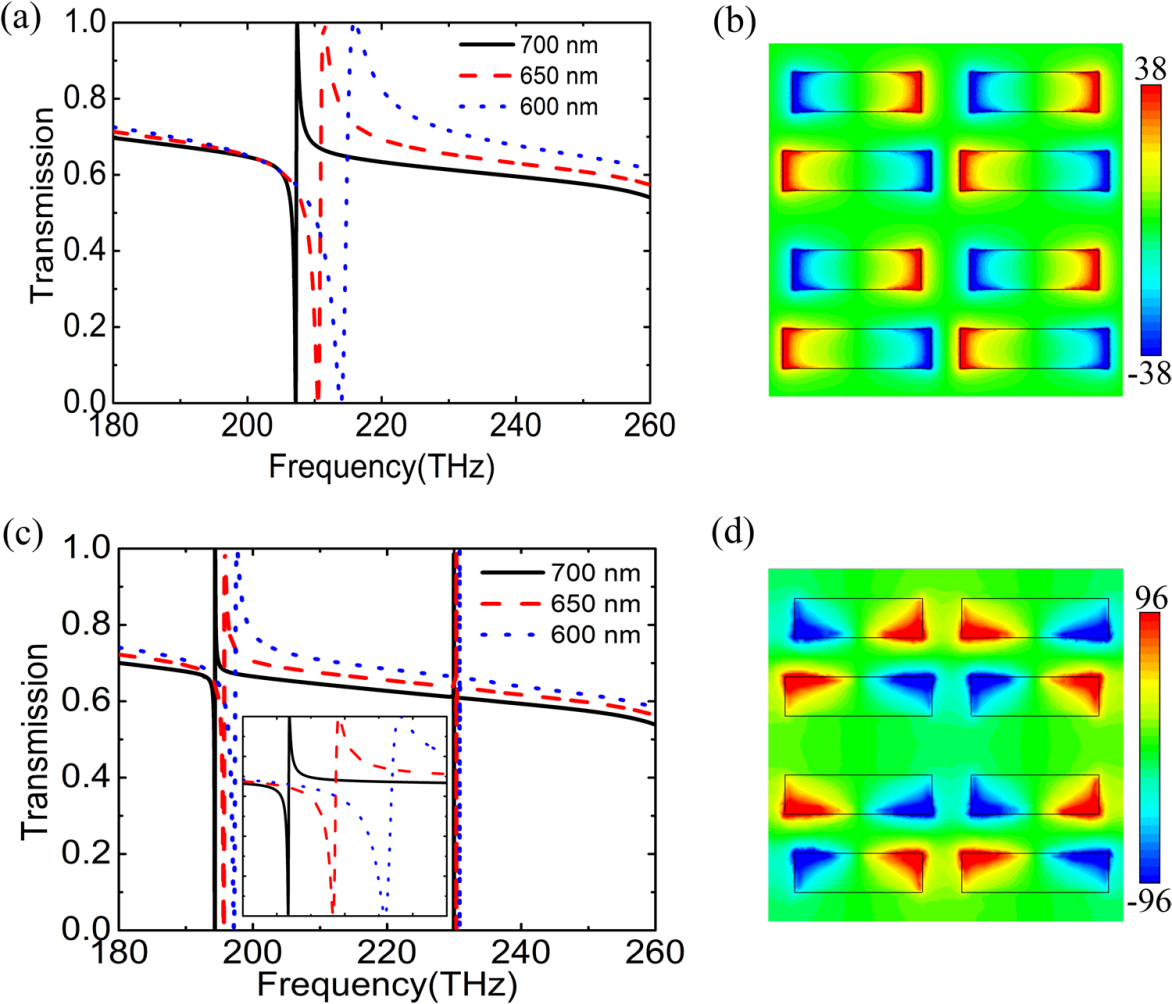
Transmission spectra of (**a**) non-flipped and (**c**) alternately flipped APBs metasurfaces with different *L*_2_. The spectra around 196 THz are zoomed in as an inset in (**c**). The normalized *z*-component of the electric field (**b**) at 210.5 THz in APBs and (**d**) at 196 THz in alternately flipped APBs for *L*_2_ = 650 nm. The field distribution is plotted in the *x*-*y* plane that is 5 nm above the top surface.

Figure 3(a) shows transmission spectra of non-flipped APBs metasurfaces (see Fig. 1(a)) with *L*_2_ = 700 nm, 650 nm and 600 nm, respectively. The transmission spectra exhibit asymmetric Fano lineshape, which originates from the excitation of anti-phased displacement currents in APBs^19^. Figure 3(b) gives the normalized *z*-component of the electric field *E*_z_/|*E*_0_| at the resonance for *L*_2_ = 650 nm, which indicates an electric quadrupole resonance. With increasing asymmetry, the corresponding Q-factor dramatically decreases, which are calculated as 925, 239 and 104 for *L*_2_ = 700 nm, 650 nm and 600 nm, respectively. The reduction of Q-factor with decreasing *L*_2_ is due to the increase of radiation loss^19^. Meanwhile, the resonant frequency of the FR shifts toward larger frequencies with increasing asymmetry because shortening of the bar length increases the excitation energy of the FR^20^.

Figure 3(c) presents calculated transmission spectra of alternately flipped APBs configuration (see Fig. 1(b)) with *L*_2_ = 700 nm, 650 nm and 600 nm, respectively. Different from the non-flipped APBs, the flipped configuration exhibits two FRs. One around 196 THz originating from the electric quadrupole mode becomes sharper. Their Q-factors are 5398, 1154 and 442 for *L*_2_ = 700 nm, 650 nm and 600 nm, respectively, which are 4~6 times larger than those of corresponding non-flipped metasurfaces. In other words, the Q-factor of the metasurface can be improved by changing configuration of unit cells, where the net dipole moment is reduced because of destructive interactions among nearest-neighbor electric dipoles. Figure 3(d) presents the normalized *z*-component of the electric field at the FR for *L*_2_ = 650 nm. It is observed that the electric field distribution of each bar is asymmetric in the *y* direction, which is attributed to combined effects of the interaction within the APBs and destructive interaction with other nearest-neighbor meta-atoms. And the combined effects result in a red-shift of the FR. The origin of the other FR around 230 THz will be explored later.

**Figure 4:**
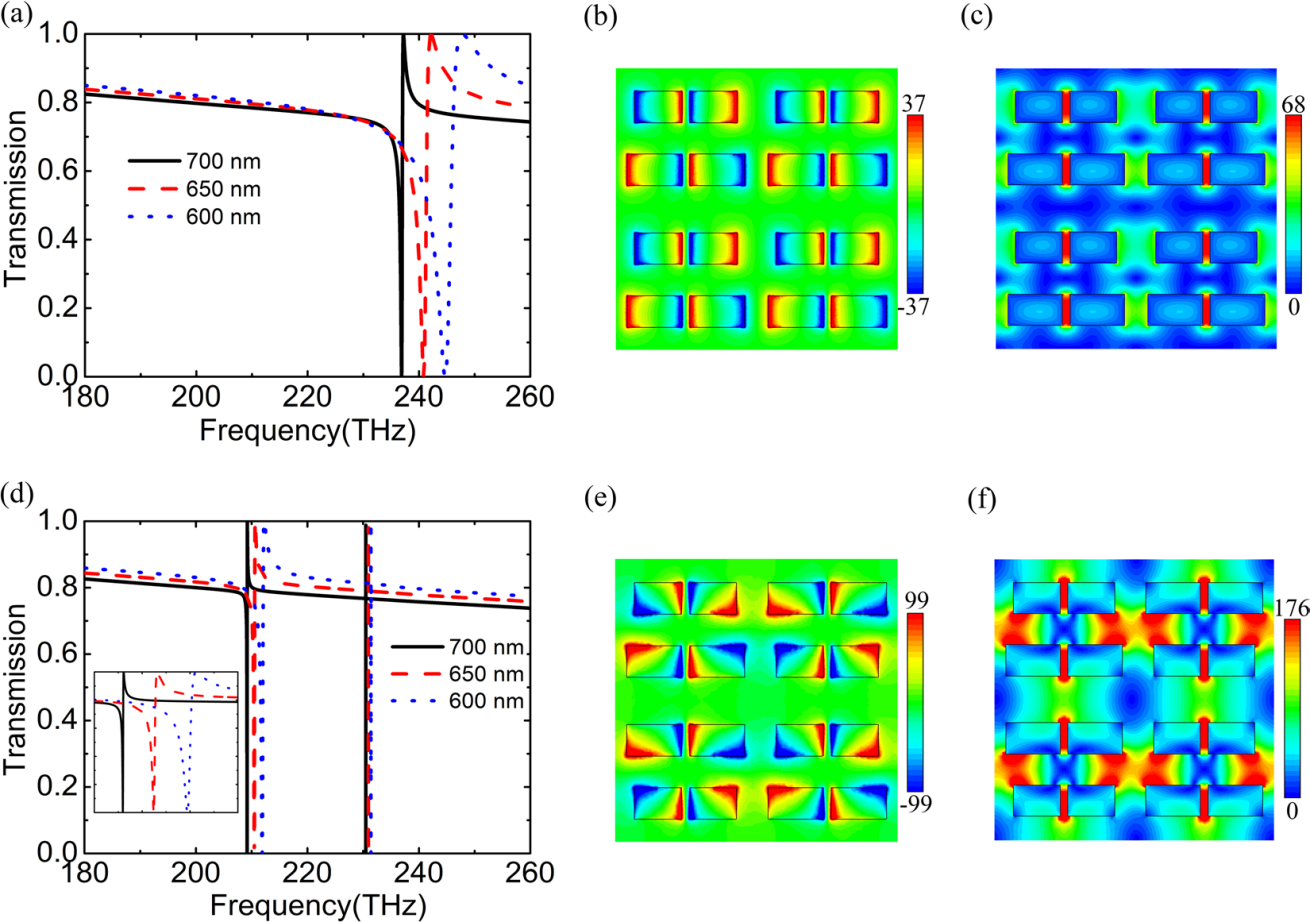
Transmission spectra of (**a**) non-flipped and (**d**) alternately flipped SAPBs metasurfaces with a gap (50 nm) for different *L*_2_. The spectra around 210 THz are zoomed in as an inset in (**d**). The normalized *z*-component of the electric field in the *x*-*y* plane that is 5 nm above the top surface of (**b**) non-flipped and (**e**) alternately flipped SAPBs, and normalized electric field in the *x*-*y* plane bisecting the bars at the FR around (**c**) 240.9 THz in SAPBs and (**f**) 210.5 THz in alternately flipped SAPBs for *L*_2_ = 650 nm.

The fields in metallic metamaterials are mainly confined at the interface, while those in all-dielectric metasurfaces are normally concentrated within the dielectric. This limits many applications of all-dielectric metasurfaces, such as biological and chemical sensing, quantum emitters, and nonlinear optics. On the other hand, nanometer-size gaps introduced in high-index dielectric resonators can improve electromagnetic fields in surrounding medium, which is due to slot waveguide^20, 25^. In order to study the gap effect, gaps are introduced in the middle of each bar. We call the structure the SAPBs (see Fig. 1(c)). Figure 4(a) presents the transmission spectra of the SAPBs metasurfaces with gaps (*g* = 50 nm) for *L*_1_ = 750 nm and *L*_2_ = 700 nm, 650 nm, 600 nm, respectively. The Q-factor remarkably decreases with the increase of asymmetry. Their Q-factors are 729, 189 and 79, respectively. Compared with corresponding structures without gaps shown in Fig. 3(a), the decrease of the Q-factor is attributed to the increase of asymmetry, and the blue shift of the resonance is due to the reduction of the effective length of the bar. Figure 4(b) shows the normalized *z*-component of the electric field at the resonance for *L*_2_ = 650 nm, which manifests the electric quadrupole mode of the SAPBs. As shown in Fig. 4(c), the strong electric field is localized in the gaps, and the maximum electric field enhancement reaches about 68. In a word, the introduction of gap decreases the Q factor somewhat. On the other hand, it concentrates the electric field within it which is favorable for enhancing the interaction between light and the surrounding medium.

Figure 4(d) shows the transmission spectra of the alternately flipped SAPBs metasurfaces (see Fig. 1(d)) with 50 nm gaps for *L*_2_ = 700 nm, 650 nm, 600 nm, respectively. The Q-factors of this FR are improved up to 9 times larger than that of corresponding non-flipped metasurfaces, which are 6537, 1422 and 562 for *L*_2_ = 700 nm, 650 nm and 600 nm, respectively. The enhancement of the Q-factor is due to destructive interaction among nearest-neighbor electric dipoles. Whereas the Q-factor decreases with reducing *L*_2_ for the increase of radiation loss. Interestingly, compared with that of alternately flipped APBs, the Q-factor becomes larger. Analogous to the redshift phenomenon in Fig. 3(c), the FR in Fig. 4(d) displays a red shift compared with Fig. 4(a). The normalized *z*-component of the electric field at 210.5 THz for *L*_2_ = 650 nm is presented in Fig. 4(e), which is akin to that of alternately flipped APBs in Fig. 3(d). The electric field is strongly localized in the gaps as well (see Fig. 4(f)). The maximum field enhancement is up to 176, which is equivalent to an enhancement factor of ~ 10^9^ for Raman applications. So the modulating of meta-atom interactions in the SAPBs not only leads to Q-factor enhancement but also further strengthens the interaction between light and the surrounding medium.

Similar to the alternately flipped APBs case, the transmission spectra also exhibit two FRs, the second FR lies at 231 THz, which is almost independent on the gap as well as *L*_2_. We ascribe it to the Rayleigh-Wood anomaly (RA), which is associated with the diffraction along grating surface. It occurs when^26^

2 $$|{\mathop{k}\limits^{\rightharpoonup }}_{0}|\sqrt{{\varepsilon }_{d}}=|{\mathop{k}\limits^{\rightharpoonup }}_{0}\,\sin \,\theta +i\frac{2\pi }{{p}_{x}}\mathop{x}\limits^{\rightharpoonup }+j\frac{2\pi }{{p}_{y}}\mathop{y}\limits^{\rightharpoonup }|$$

where *ε*_*d*_ is the dielectric constant of the background material (vacuum in our work), ${\mathop{k}\limits^{\rightharpoonup }}_{0}$ is the wave vector of the incident wave in vacuum, *p*_*x*_ (*p*_*y*_) represents the period along *x* (*y*) direction, *i* and *j* are integers defining the different diffraction orders, respectively. The RA (±1, ±1) frequency is 235.7 THz which approximately agrees with that of the second FRs in alternately flipped metasurfaces. The Fano lineshape originates from the interaction between the electric dipole and the light diffracted parallel to grating surface, which can find applications in refractive index sensing^27^.

**Figure 5:**
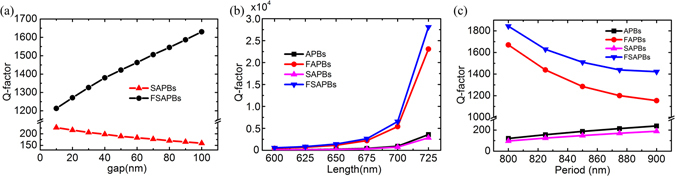
(**a**) Q-factor of the FR as a function of gap’s width in SAPBs and FSAPBs with *L*_2_ = 650 nm. (**b**) Q-factor of the FR as a function of *L*_2_. (**c**) Q-factor of the FR as a function of *p*.

Contrary to the SAPBs case, the Q-factor of alternately flipped SAPBs (FSAPBs) increases with the gap, as shown in Fig. 5(a). This is because of the fact that the offset of the electric dipole moment in the *y* direction further reduces the radiation loss. Figure 5(b) presents the Q-factor of the FRs as a function of *L*_2_ for non-flipped APBs/SAPBs, alternately flipped APBs (FAPBs) and FSAPBs. The Q-factors of FRs in these four metasurfaces are enhanced with increasing *L*_2_, which originates from suppressing the radiation loss. In metasurfaces with alternately flipped configuration, Q-factors are much larger than those of corresponding metasurfaces with non-flipped configuration. The Q-factors are enhanced up to 6.5 and 9.8 times for APBs and SAPBs with *L*_2_ = 725 nm, respectively. The Q-factor could be as large as the order of 10^8^ in alternately flipped configurations when *L*_2_ = 749.9 nm (not shown here). Figure 5(c) shows the Q-factor of the FRs as a function of period *p*_*x*_ = *p*_*y*_ = *p* for APBs, SAPBs, FAPBs and FSAPBs with *L*_1_ = 750 nm and *L*_2_ = 650 nm. With the decrease of *p*, the constructive interaction in non-flipped configurations is strengthened, leading to a decrease of the Q-factor of the FRs, while the destructive interaction in alternately flipped configurations is also strengthened, leading to a quick increase of the Q-factor of the FRs. Therefore, the destructive coupling effect is an effective approach to achieve high Q-factor for all-dielectric metasurfaces, besides the structural design widely reported in literatures.

**Figure 6:**
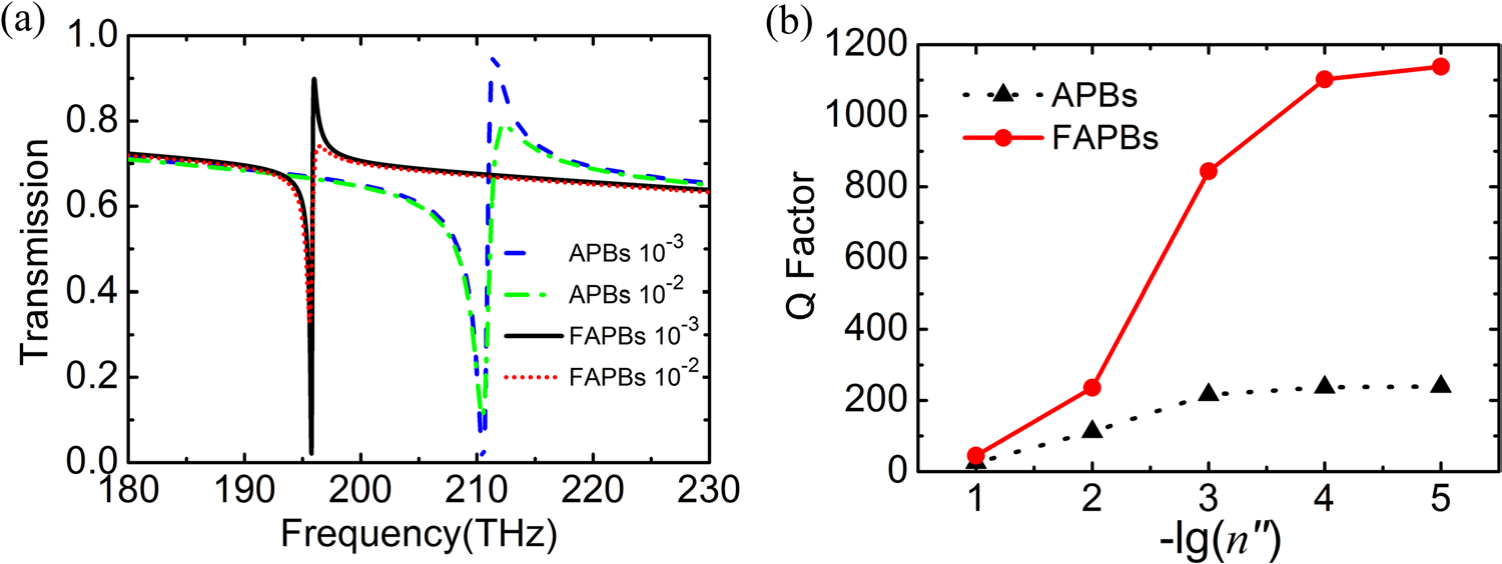
Effects of absorption loss on Q-factor of the FR in APBs and alternately FAPBs metasurfaces with *L*_1_ = 750 nm and *L*_2_ = 650 nm. (**a**) Transmission spectra for *n*″ = 10^−3^ and 10^−2^, respectively. (**b**) Q-factor of the FR as a function of −lg(*n*″).

The influence of absorption loss on the Q-factor of the FR in the APBs and alternately FAPBs metasurfaces is further investigated, as shown in Fig. 6. Here, the refractive index of Si is described as *n*_Si_ = 3.5 + *in″*. Figure 6(a) presents transmission spectra of the APBs and alternately FAPBs with *L*_1_ = 750 nm and *L*_2_ = 650 nm for *n″* = 10^−3^ and 10^−2^, respectively. The absorption loss reduces the spectral contrast and slightly increases the difference of the frequency at transmission peak and dip, which reveals a decrease of the Q-factor with increasing of the dielectric loss. Figure 6(b) shows the Q-factor of the FR as a function of -lg(*n″*). With decreasing -lg(*n″*), i.e. the increase of absorption loss, the Q-factor of the FR in alternately FAPBs is more rapidly attenuated than that in APBs. In other words, the less the absorption loss is, the more the Q-factor of FRs can be enhanced by introducing alternately flipped configuration. This can account for the results of metallic metamaterials, in which the Q-factor is slightly improved by changing configuration^15, 23^.

## Conclusion

In conclusion, we numerically investigated the high-Q FRs in all-dielectric metasurfaces. By introducing the alternately flipped configurations, the Q-factor of the FR can be markedly enhanced, which is attributed to the destructive interaction among nearest-neighbor meta-atoms. The destructive interaction reduces the net electric dipole moment and suppresses the radiation loss. The offset of the *y*-component of the electric dipole moment further improves the Q-factor in the alternately flipped SAPBs, where the electric field is strongly located in the gap and the Q-factor exceptionally increases with the gap. Furthermore, the less the absorption loss is, the more the Q-factor of FRs can be enhanced.

## Methods

The Si metasurfaces are initially supposed to be lossless with a refractive index of *n* = 3.5, and the material loss is considered further (*n*_Si_ = 3.5 + *in″*). The surrounding medium is supposed to be air (*n* = 1). The transmission spectra and electric field distributions are calculated using the frequency-domain finite element method with a tetrahedral mesh. The periodic boundary conditions for each unit cell are used in the *x* and *y* axes, and open boundary condition is used in the *z* axis (propagation direction). At least 10 mesh steps per wavelength and adaptive tetrahedral mesh refinement are used to ensure the accuracy of the calculated results.

The Q-factor is extracted from the transmission spectra of metasurfaces, which is expressed as *Q* = *f*/Δ*f*, where *f* is the resonant frequency of the FR and Δ*f* is defined as the difference of the frequencies at the transmission peak and dip.

## References

[1] Luk’yanchuk B (2010). The Fano resonance in plasmonic nanostructures and metamaterials. Nat Mater.

[2] Cao W (2012). Low-loss ultra-high-Q dark mode plasmonic Fano metamaterials. Opt Lett.

[3] Liu SD (2016). Polarization-Independent Multiple Fano Resonances in Plasmonic Nonamers for Multimode-Matching Enhanced Multiband Second-Harmonic Generation. Acs Nano.

[4] Ye J (2012). Plasmonic Nanoclusters: Near Field Properties of the Fano Resonance Interrogated with SERS. Nano Lett.

[5] Zheludev NI, Prosvirnin SL, Papasimakis N, Fedotov VA (2008). Lasing spaser. Nat Photonics.

[6] Manjappa M, Srivastava YK, Cong LQ, Al-Naib I, Singh R (2017). Active Photoswitching of Sharp Fano Resonances in THz Metadevices. Adv Mater.

[7] Zu S, Bao YJ, Fang ZY (2016). Planar plasmonic chiral nanostructures. Nanoscale.

[8] Fang ZY (2012). Plasmon-Induced Doping of Graphene. Acs Nano.

[9] Lu H, Liu XM, Mao D, Wang GX (2012). Plasmonic nanosensor based on Fano resonance in waveguide-coupled resonators. Opt Lett.

[10] Gupta M, Srivastava YK, Manjappa M, Singh R (2017). Sensing with toroidal metamaterial. Appl Phys Lett.

[11] Zhang S, Genov DA, Wang Y, Liu M, Zhang X (2008). Plasmon-induced transparency in metamaterials. Phys Rev Lett.

[12] Hao F (2008). Symmetry Breaking in Plasmonic Nanocavities: Subradiant LSPR Sensing and a Tunable Fano Resonance. Nano Lett.

[13] Li GZ, Li Q, Wu LJ (2015). Double Fano resonances in plasmonic nanocross molecules and magnetic plasmon propagation. Nanoscale.

[14] Moritake Y, Kanamori Y, Hane K (2014). Experimental demonstration of sharp Fano resonance in optical metamaterials composed of asymmetric double bars. Opt Lett.

[15] Gupta M, Singh R (2016). Toroidal versus Fano Resonances in High Q planar THz Metamaterials. Adv Opt Mater.

[16] Evlyukhin AB, Reinhardt C, Seidel A, Luk’yanchuk BS, Chichkov BN (2010). Optical response features of Si-nanoparticle arrays. Phys Rev B.

[17] Jahani S, Jacob Z (2016). All-dielectric metamaterials. Nat Nanotechnol.

[18] Zhao WY, Leng XD, Jiang YY (2015). Fano resonance in all-dielectric binary nanodisk array realizing optical filter with efficient linewidth tuning. Opt Express.

[19] Zhang JF, MacDonald KF, Zheludev NI (2013). Near-infrared trapped mode magnetic resonance in an all-dielectric metamaterial. Opt Express.

[20] Zhang JF, Liu W, Zhu ZH, Yuan XD, Qin SQ (2014). Strong field enhancement and light-matter interactions with all-dielectric metamaterials based on split bar resonators. Opt Express.

[21] Al-Naib I (2012). Excitation of a high-Q subradiant resonance mode in mirrored single-gap asymmetric split ring resonator terahertz metamaterials. Appl Phys Lett.

[22] Al-Naib I, Yang YP, Dignam MM, Zhang WL, Singh R (2015). Ultra-high Q even eigenmode resonance in terahertz metamaterials. Appl Phys Lett.

[23] Moritake Y, Kanamori Y, Hane K (2015). Enhanced quality factor of Fano resonance in optical metamaterials by manipulating configuration of unit cells. Appl Phys Lett.

[24] Volakis, J. L., Chatterjee, A. & Kempel, L. C. *Finite element method electromagnetics: antennas, microwave circuits, and scattering applications*. Vol. 6 (John Wiley & Sons, 1998).

[25] Yang YM, Kravchenko II, Briggs DP, Valentine J (2014). All-dielectric metasurface analogue of electromagnetically induced transparency. Nat Commun.

[26] Gao H (2009). Rayleigh anomaly-surface plasmon polariton resonances in palladium and gold subwavelength hole arrays. Opt Express.

[27] Shen Y (2013). Plasmonic gold mushroom arrays with refractive index sensing figures of merit approaching the theoretical limit. Nat Commun.

